# Validation of a simple risk stratification tool for COVID-19 mortality

**DOI:** 10.3389/fmed.2022.1016180

**Published:** 2022-10-11

**Authors:** Angela Horvath, Theresa Lind, Natalie Frece, Herbert Wurzer, Vanessa Stadlbauer

**Affiliations:** ^1^Medical University of Graz, Graz, Austria; ^2^Center for Biomarker Research in Medicine (CBmed), Graz, Austria; ^3^Department of Internal Medicine, State Hospital Graz II, Graz, Austria

**Keywords:** COVID-19, risk score, prediction, validation, mortality

## Abstract

Risk prediction is an essential part of clinical care, in order to allocate resources and provide care appropriately. During the COVID-19 pandemic risk prediction became a matter of political and public debate as a major clinical need to guide medical and organizational decisions. We previously presented a simplified risk stratification score based on a nomogram developed in Wuhan, China in the early phase of the pandemic. Here we aimed to validate this simplified risk stratification score in a larger patient cohort from one city in Austria. Age, oxygen saturation, C-reactive protein levels and creatinine levels were used to estimate the in-hospital mortality risk for COVID-19 patients in a point based score: 1 point per age decade, 4 points for oxygen saturation <92%, 8 points for CRP > 10 mg/l and 4 points for creatinine > 84 μmol/l. Between June 2020 and March 2021, during the “second wave” of the pandemic, 1,472 patients with SARS-CoV-2 infection were admitted to two hospitals in Graz, Austria. In 961 patients the necessary dataset to calculate the simplified risk stratification score was available. In this cohort, as in the cohort that was used to develop the score, a score above 22 was associated with a significantly higher mortality (*p* < 0.001). Cox regression confirmed that an increase of one point in the risk stratification score increases the 28-day-mortality risk approximately 1.2-fold. Patients who were categorized as high risk (≥22 points) showed a 3–4 fold increased mortality risk. Our simplified risk stratification score performed well in a separate, larger validation cohort. We therefore propose that our risk stratification score, that contains only two routine laboratory parameter, age and oxygen saturation as variables can be a useful and easy to implement tool for COVID-19 risk stratification and beyond. The clinical usefulness of a risk prediction/stratification tool needs to be assessed prospectively (https://www.cbmed.at/covid-19-risk-calculator/).

## Introduction

During the COVID-19 pandemic hospital and ICU beds were scarce resources and hospital capacities became a matter of political and public debate. Accurate risk stratification for patients with COVID-19 admitted to the hospital therefore is a major clinical need to guide medical and organizational decisions. However, reliable risk stratification tools to address this problem were and are still lacking. A multitude of studies aimed to predict the risk of severe disease and mortality as early as possible in the course of COVID-19 infections. These risk stratification attempts were ranging from complex biomarker studies that warrant resource intensive research settings (1) to relatively easy to obtain scores that require only routine laboratory data from hospital admission (2). Also non-laboratory markers such as arterial stiffness (3), lung sonography (3), primary care data (4) or the “repurposing” of established risk stratification scores in general hospital populations were studied (5). The methods to combine biomarker into risk prediction score can range from single-parameter to multiple-parameter and aggregate weighted systems (6). The methods to create and validate such risk scores range from traditional biostatistical approaches to novel artificial intelligence models (7). However so far, none of these scores for COVID-19 disease severity prediction made its way to clinical routine.

Already early in the course of the pandemic, data from a large dataset of the first wave of the pandemic in Wuhan/China showed that routine laboratory markers available at admission could accurately predict COVID-19 disease outcome (8). We aimed to validate this score in a real-world dataset for a European cohort. The validation was successful, however, we noticed that the score, despite being based on routine laboratory parameters, was rather complex to calculate and outside a clinical study setting many missing data would further impaired clinical applicability. We therefore took this nomogram as a basis and developed a simple and easy to calculate risk stratification score. Our score stratifies the mortality risk of hospitalized patients with COVID-19 based on only four variables: age, oxygen saturation, C-reactive protein and creatinine (9). Ding et al. tested the robustness of our simplified model in their original cohort from Wuhan and found that our simplified predictive model can predict 28-day mortality well, however with a somewhat reduced accuracy (10).

We now set out to test the robustness of our simplified score and the initial nomogram from Ding et al. again during the second wave of the pandemic between June 2020 and March 2021 in Graz, Austria.

## Methods

We retrospectively collected demographic and laboratory data as well as in-hospital mortality from all patients (without age limitations) hospitalized at either the University Hospital Graz or the State Hospital Graz II between June 2020 and March 2021. Patients' information was extracted using the ICD10 code U07.1. SARS-CoV-2 infection was manually verified by 2 independent investigators in each case by the documentation of a result of a positive SARS-CoV-2 PCR. The study was approved by the institutional review board (32–431 ex 19/20), informed consent was waived due to the retrospective nature of the study and the study was registered at clinicaltrials.gov (NCT04420637).

### Risk stratification

Age, oxygen saturation, C-reactive protein levels and creatinine levels were used to estimate the in-hospital mortality risk for COVID-19 patients, as previously proposed in (9): 1 point per age decade, 4 points for oxygen saturation <92%, 8 points for CRP > 10 mg/l and 4 points for creatinine > 84 μmol/l. A score of 22 or higher indicates a significantly increased mortality risk.

Parameters for the risk score calculation were assessed at the day of admission (+1 day) if the patient was admitted with or because of a SARS-CoV-2 infection, or as the day of diagnosis (+1 day), if patients contracted SARS-CoV-2 during an unrelated hospital stay. In case, a parameter was assessed more than once within the defined time period, the earliest documented value was used.

### Statistical analysis

The predictive merit of the risk stratification score was validated using different approaches. First, AUROC analysis was performed to test whether the risk stratification score can accurately predict which patients died within the defined time period of 7, 14, 21 or 28 days after admission/diagnosis. Next, the previously published cutoff of 22 was used to categorize the patients in a high risk and a low risk group. Kaplan Meier curves and log rank tests were performed to test whether patients in the high risk group actually have a significantly higher mortality risk compared to patients in the low risk group. The cutoff of 22 was further validated by comparison to a cutoff optimized to the data set at hand. A Monte-Carlo simulation was run to find the cutoff with the highest accuracy for 28-day mortality. In this simulation, the data set was randomly split into a training set (70% cases) and a test set (30% of cases), every possible cutoff (i.e. every integer between 1 and 25) was applied and the overall accuracy in the training set was compared. The best performing cutoff was then applied to the test set and its accuracy was documented. This sequence was repeated 100.000 times and the modus of the three best performing cutoffs was defined as the optimized cutoff for the data set at hand. Chi-square test was used to compare the proportion of accurate predictions between the proposed and the optimized cutoff.

Cox regression was used to estimate the hazard ratio for the risk stratification score, for the categorization as high or low risk group, as well as for each parameter of the score individually.

Analysis was performed with R and R-Studio using the packages “tidyverse”, “readxl”, “ggpubr”, “data.table”, “lubridate”, “caret”, “survival”, “survminer”, “pROC”, “ROCR” and “foreign”.

## Results

During the second wave of the pandemic, 1511 individual patients were hospitalized with the diagnosis code U07.1 for COVID-19 infection. After exclusion of 39 patients with no verifiable SARS-CoV-2 infection, from the remaining 1,472 patients, in 961 patients the necessary dataset for our simplified score was available whereas the full nomogram from Ding et al. (8) could only be calculated for 171 patients because of missing data. Compared to the cohort used to establish the risk stratification score, the patients analyzed in this study were younger, were less likely to have reduced oxygen saturation, had higher creatinine levels and consequently also had a higher risk stratification score. Mortality and C-reactive protein levels was comparable between the study cohorts. See Table 1 for details.

**Table 1:** Patient characteristics of the validation cohort and the patients on which the risk stratification score was based (patients 3–6/2020).

**Parameter**	**Second wave (6/2020–3/2021; *n* = 961)**	**First wave (3–6/2020; *n* = 243)**	**Standardized difference**
**Age (years)**	**68.6** **±18.5**	**74.9** **±14.5**	**0.38**
Female (%)	437 (45.5)	108 (44.4%)	−0.02
Communally acquired infection (%)	859 (89.4)	na	na
Oxygen saturation (%)	92.8 ± 5.8	92.6 ± 5.2	−0.03
**oxygen saturation** ** <92%** (%)	**268 (27.9)**	**97 (39.9)**	–**0.26**
C-reactive protein (mg/l)	83.7 ± 80.2	80.0 ± 78.3	−0.05
C-reactive protein over 10 mg/l (%)	818 (85.1)	203 (83.5)	0.04
**Creatinine (μmol/l)**	**123.2** **±121.4**	**110.9** **±107.8**	**1.44**
Creatinine over 84 μmol/l (%)	572 (59.5)	133 (54.7)	0.10
**Risk stratification score**	**16.7** **±5.1**	**18.4** **±5.0**	**0.33**
**Risk stratification score** **≥22** (%)	**134 (13.9)**	**70 (28.8)**	–**0.37**
Mortality			
Died within 7 days of hospitalization (%)	99 (10.3)	20 (8.2)	0.07
Died within 14 days of hospitalization (%)	170 (17.7)	56 (23.0)	−0.13
Died within 21 days of hospitalization (%)	196 (20.4)	62 (25.5)	−0.12
Died within 28 days of hospitalization (%)	221 (22.0)	63 (25.9)	−0.09

Comparability of the patient groups is shown as standardized difference. An absolute value below 0.2 signifies good agreement.

Bold values indicate parameters without good agreement.

### Risk stratification score validation

AUROC analysis confirmed that the proposed risk stratification score is predictive of 7, 14, 21 and 28-day mortality of hospitalized COVID-19 patients in the new cohort (6/2020–3/2021). Details are given in Table 2.

**Table 2:** AUROC analysis of risk stratification score for 7, 14, 21 and 28-day mortality.

	**C-value**	**95% confidence interval**
7-day mortality	0.75	0.71–0.80
14-day mortality	0.75	0.71–0.79
21-day mortality	0.74	0.70–0.78
28-day mortality	0.73	0.69–0.77

**Figure 1:**
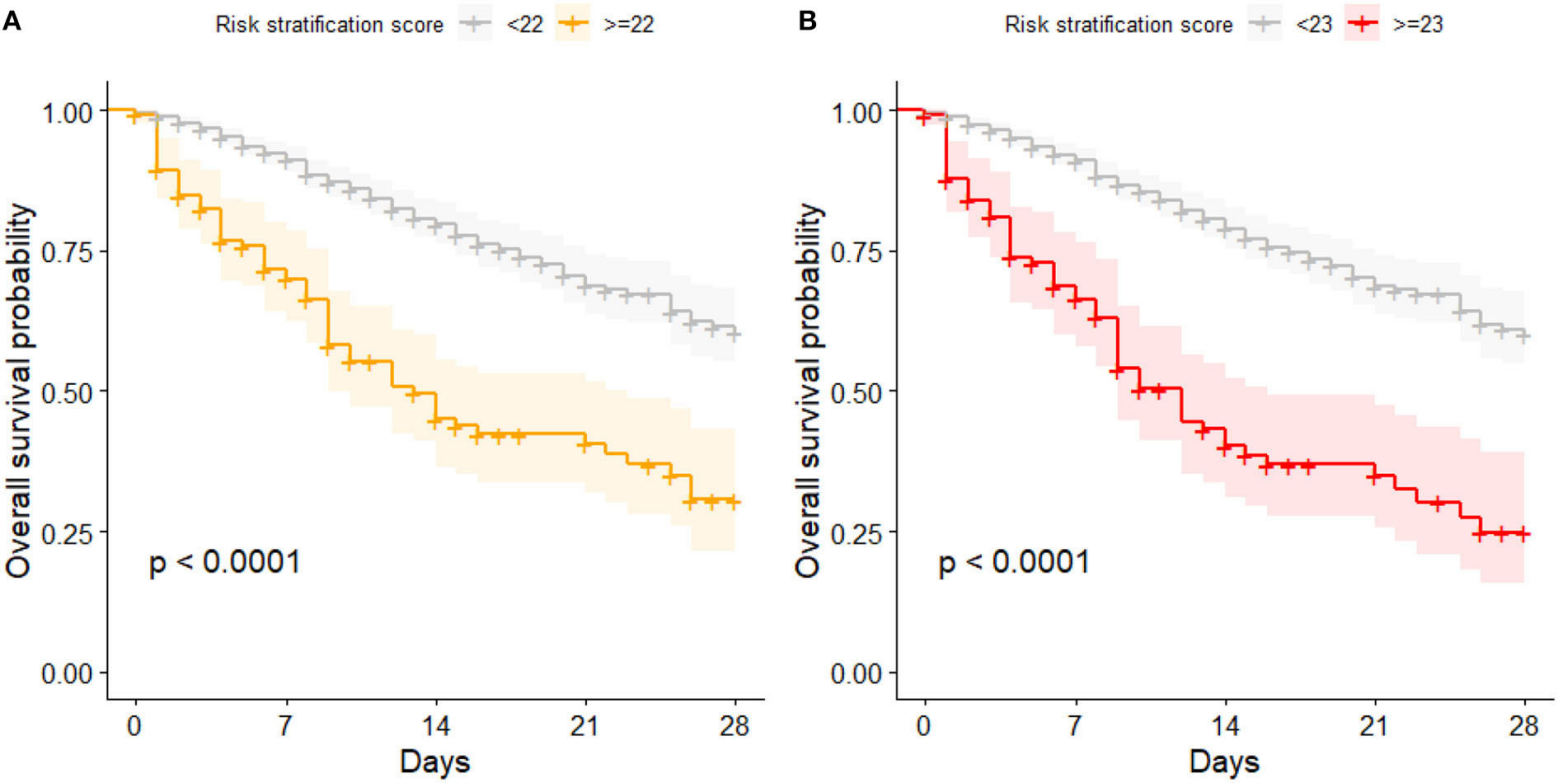
Kaplan Meier curves for patients with high and low risk of mortality; **(A)** categorization based on the proposed cutoff of 22 points; **(B)** categorization based on the cutoff optimized for this data set (23 points).

When the risk stratification score was proposed on the cohort from 3 to 6/2020, a score of 22 or above indicated an increased COVID-19-related in-hospital mortality risk. Also in this study, patient with a score of 22 or above showed a significantly higher mortality risk compared to patients with a score below 22 (*p* < 0.001). However, the optimized cutoff with the highest overall accuracy for the present cohort was 23. It showed a slightly better accuracy, but there was no significant difference in the number of accurately classified patients compared to the cutoff of 22 points (85.5 vs. 83.7%, respectively, *p* = 0.3). Figure 1 compares the Kaplan Meier curves for both cutoffs.

### Estimation of hazard ratios

Cox regression confirmed that an increase of one point in the risk stratification score increases the 28-day-mortality risk approximately 1.2-fold (details are given in Table 3, information about 7, 14, and 21-day mortality is given in the supplements). Also patients who were categorized as high risk (≥22 points) showed a 3–4 fold increased mortality risk, depending on the observation period. Accordingly, all parameters of the risk stratification score were associated with increased mortality risk to varying degrees. Interestingly, while C-reactive protein levels were associated with increased mortality risk, the categorization of high and low levels as initially proposed by the risk stratification score was not a constant significant predictor. Patients showed high levels of C-reactive protein irrespective of the outcome (see Supplementary Figure 1). Although CRP levels were comparable in the initial publication describing the patients from the first wave of the pandemic (3–6/2020), its predictive merit could not be reproduced in patients from the later phase (second wave, 6/202–3/2021). To account for superimposed bacterial infections already at admission, patients with leucocytosis (leucocyte count >11.3 G/l) were temporarily excluded from analysis, however it did not improve the prediction based on increased C-reactive protein levels.

**Table 3:** Hazard ratios for 28-day mortality of the risk stratification score, its components and categorizations.

**Predictor**	**Hazard ratio**	**95% confidence interval**	***p*-value**
Age	1.056	1.043–1.068	<0.001
Age points	1.682	1.505–1.880	<0.001
Oxygen saturation	0.9501	0.9366–0.9638	<0.001
Oxygen saturation <92%	2.048	1.562–2.685	<0.001
C-reactive protein	1.003	1.002–1.005	<0.001
C-reactive protein >10 mg/l	1.614	0.9952–2.617	0.052
Creatinine	1.106	1.041–1.176	0.001
Creatinine >84 μmol/l	1.702	1.260–2.299	<0.001
Risk stratification score	1.179	1.133–1.226	<0.001
Risk stratification score >22	3.084	2.317–4.105	<0.001
Risk stratification score >23	3.550	2.640–4.775	<0.001

### Comparison of the risk stratification score with the nomogram by Ding et al.

**Figure 2:**
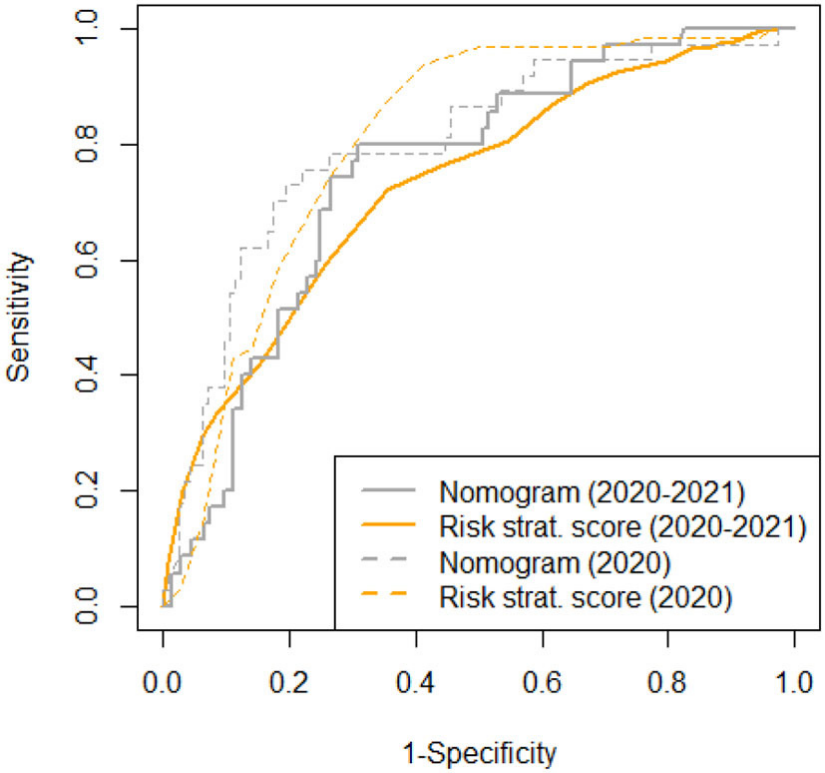
AUROC for the risk stratification score and the nomogram (8) for all available data sets (*n* = 961 and *n* = 177, respectively) compared to previously published data (9).

In the presented validation cohort, also the nomogram from Ding et al. predicted 28-day in-hospital mortality, whereby an increase of one point is associated with a 1.007-fold (95%CI: 1.003–1.012; *p* = 0.002) increase in mortality risk. Comparing areas under the receiver operated characteristics curve (AUROC) in all available data sets, the full nomogram showed only a slightly but not significantly better prediction when compared to our simplified risk stratification score (AUC-difference: −0.019; *p* = 0.7) (Figure 2). However, full datasets necessary to apply the nomogram from Ding et al. were available for significantly less patients in comparison to our simplified risk stratification score (12 vs. 65%, *p* < 0.001).

## Discussion

Triage management plays important roles in hospitalized patients for disease severity stratification and medical burden analysis. Although risk prediction scores have been extensively researched for many acute and chronic diseases, there was an urgent need to adapt and validate risk prediction scores in COVID-19 disease (11).

While the use of complex research biomarkers, such as metabolomic analyses (12) or deep immune phenotyping (13), is of great interest to understand the pathophysiology of this disease better, especially in vulnerable patient groups, the clinical applicability of such complex biomarkers and scores is currently limited due to lack of availability and high costs. A review of 76 different coring systems, ranging from existing scores to newly developed scores, artificial intelligence algorithms and novel biomarker came to the conclusion that all of these scores have limitations but that the combination of single laboratory parameters may have the greatest potential for implementation (14).

Identification of an easily applied and valid evidence-based clinical risk stratification tool is therefore an unmet clinical need that we tried to fulfill. We started from the highly predictive but rather complex nomogram created by Ding et al., that was developed based on the results of a multivariate analysis that contained an extensive routine laboratory parameter workup including full blood count, liver and renal function tests, cardiac troponin I, lactate dehydrogenase, CRP, procalcitonin and cytokines as well as hepatitis B-related antigen or antibodies, and hepatitis C-related antibodies. In addition, age and the findings from a CT scan of the chest were included. From that dataset, 8 laboratory tests (lymphocyte count, platelets, CRP, D-dimer, creatinine, cardiac troponin I, aspartate aminotransferase, direct bilirubin) as well as two clinical parameters (age and severity of pneumonia) were derived and the nomogram was developed. In our initial publication we were able to first of all validate the predictive power of the parameters identified in a Chinese cohort and in a next step we were able to reduce the number of parameters to two clinical and two laboratory parameters without losing diagnostic accuracy (9). In an effort to enhance the accuracy with parameters not included in the nomogram, we also considered comorbidities as potential outcome predictors: First we evaluated 23 comorbidities derived from the Charlson Comorbidity Index separately for their association with COVID19-related outcome. We observed that obesity, cancer, liver disease, arterial hypertension, heart failure and peripheral arterial disease were not associated with outcome. Leukemia, lymphoma, metastatic cancer, AIDS, hemiplegia, connective tissue diseases, gastrointestinal ulcera and inflammatory bowel disease had a low prevalence and therefore did not contribute significantly to outcome prediction in our study population. Dementia, Morbus Parkinson, kidney diseases, diabetes mellitus, coronary artery disease, myocardial infarction, cardiac arrhythmias, cerebrovascular diseases and chronic lung diseases were significantly associated with outcome but highly dependent on age and therefore could not contribute significantly to outcome prediction in a model that strongly featured age as a main predictor. We also used the point score derived from Charlson Comorbidity Index (original, updated and age-adjusted) but age was such a strong factor in both cohorts, that there was no additional benefit in adding comorbidities to the score. Therefore, age, oxygen saturation, C-reactive protein and creatinine were finally implemented in a weighted sums score to predict 28-day mortality. Our validation and the validation performed by Ding et al. (10) shows the robustness of our simplified risk calculation model over different times and across continents. Although the original nomogram from Ding et al. (8) has a slightly better performance, our real-life dataset shows that under routine working conditions outside a study setting, the full dataset necessary to apply the nomogram from Ding et al. was available only from a minority (12%) of patients in the Austrian cohort. In comparison, the simplified risk stratification score was retrospectively calculable in 65% of patients. The cohort characteristics between the first and the second wave of the pandemic differed. In the second wave, patients were younger, had less severe pneumonia as indicated by oxygen saturation <92%, but higher creatinine levels. Despite these differences, the risk stratification score worked equally well with the same cut-off. This indicates the robustness of our model and even allows the hypothesis that this score may be useful outside of COVID-19.

An ideal clinical score requires simplicity of calculation, not too many variables that need to be easily available, independent validation, and should provide clinical detection as early as possible (15). For the field of cardiovascular risk prediction, it is known that factors influencing the successful implementation of risk scoring are related to clinical setting and healthcare system (resources, priorities, practice culture and organization), users (attributes and interactions between users) and the specific risk tool (characteristics, perceived role and effectiveness) (16). We believe that our COVID-19 risk stratification score fulfills the requirements that would allow successful implementation. Also, from a cost perspective, a score that only requires two laboratory variables instead of eight, also has an advantage, especially in resource limited settings. Our simplified COVID-19 risk stratification score can also be easily calculated without any technical help, however, especially in the younger generation of physicians, online/mobile applications are frequently used and highly accepted in clinical care (17). Therefore we offer our score as an open source online calculator (https://www.cbmed.at/covid-19-risk-calculator/). Ideally this calculator can be implemented in electronic health records, allowing automated calculation of the risk score from data obtained at hospital entry in each patient with COVID-19 infection.

The next step for assessing the clinical usefulness of a risk prediction/stratification tool would be to assess the score prospectively and draw clinical conclusions from the result. This has not been performed yet with our score. Such an undertaking also raises ethical questions: in resource rich settings, a high score, indication a high risk for mortality, would most likely trigger the allocation of resource to this patient (intensive monitoring, early referral to intermediate or intensive care). However, in resource-restricted settings, the opposite may be the case—people with a predicted adverse outcome may be withheld from intensive care treatment in triage situations. Triage here refers to situations where different patient priority groups are established in order to distribute scarce health resources. An in depth review on the literature of triage in the COVID-19 pandemic came to the conclusion that there is consensus to rely on medical prognosis, maximizing lives saved, justice as fairness and non-discrimination (18). Several open points were identified, such as the need for improved outcome predictions, possibly aided by artificial intelligence, the development of participatory approaches to drafting, assessing and revising triaging protocols and the need to learn from experiences with implementation of guidelines with a view to continuously improve decision-making (18).

Our study has some limitations: due to the retrospective nature of our study missing data led to the exclusion of 12% of the datasets. The inclusion of only two centers still warrants further validation of the score in multicenter datasets from different regions to test the robustness across different health care systems. We also did not analyze the impact of different non-specific or specific therapies administered during COVID-19 infection on outcome and on the performance of our score. However, the fact that we could validate the score in the second wave of COVID-19, where treatment with steroids and remdesivir was already well established, as opposed to the first wave, is reassuring that the score is robust.

In conclusion we propose a simple risk stratification score based on age, oxygen saturation (as an indicator for severity of pneumonia), creatinine and C-reactive protein, to differentiate between patients with high and low mortality risk from COVID-19 when admitted to the hospital.

## Data availability statement

The raw data supporting the conclusions of this article will be made available by the authors, without undue reservation.

## Ethics statement

The studies involving human participants were reviewed and approved by Ethikkommission Medizinische Universität Graz, Auenbruggerplatz 2, 8036 Graz, IRB00002556. Written informed consent for participation was not required for this study in accordance with the national legislation and the institutional requirements.

## Author contributions

TL, NF, and HW collected data. AH and VS analyzed the data and wrote the manuscript. All authors contributed to the article and approved the submitted version.

## Funding

The analysis of the dataset was funded by the Center for Biomarker Research in Medicine (CBmed), a COMET K1 center funded by the Austrian Research Promotion Agency (Project 3.23).

## Conflict of interest

The authors declare that the research was conducted in the absence of any commercial or financial relationships that could be construed as a potential conflict of interest.

## Publisher's note

All claims expressed in this article are solely those of the authors and do not necessarily represent those of their affiliated organizations, or those of the publisher, the editors and the reviewers. Any product that may be evaluated in this article, or claim that may be made by its manufacturer, is not guaranteed or endorsed by the publisher.
