# Supplementary material for: Validation of a simple risk stratification tool for COVID-19 mortality
Source: Front Med (Lausanne). 2022 Oct 11;9:1016180. doi: 10.3389/fmed.2022.1016180 (PMC9592707; doi:10.3389/fmed.2022.1016180)
Supplement: Supplementary file 1 [file Data_Sheet_1.pdf]

Supplementary Information

Validation of a simple risk stratification tool for COVID-19 mortality

Angela Horvath,[1,2] Theresa Lind,[1] Natalie Frece,[1] Herbert Wurzer[3], Vanessa Stadlbauer[1,2]
[1]Medical University of Graz, Graz, Austria
[2]Center for Biomarker Research in Medicine (CBmed), Graz, Austria
[3]Department of Internal Medicine, State Hospital Graz II, Graz, Austria

Supplementary Tables

TableS1: Hazard ratios for 7-day mortality of the risk stratification score, its components and categorizations.

Predictor	Hazard Ratio	95% Confidence Interval	p-value
Age	1.067	1.048-1.086	<0.0001
Age points	1.824	1.545-2.153	<0.0001
Oxygen saturation	0.9344	0.9159-0.9533	<0.0001
Oxygen saturation <92%	2.379	1.604-3.528	<0.0001
C-reactive protein	1.005	1.003-1.007	<0.0001
C-reactive protein >10mg/l	2.396	1.049-5.473	0.038
Creatinine	1.175	1.088-1.269	<0.0001
Creatinine >84µmol/l	2.907	1.763-4.793	<0.0001
Risk stratification score	1.265	1.189-1.346	<0.0001
Risk stratification score >22	4.134	2.757-6.199	<0.0001
Risk stratification score >23	4.569	3.018-6.919	<0.0001

Table S2: Hazard ratios for 14-day mortality of the risk stratification score, its components and categorizations.

Predictor	Hazard Ratio	95% Confidence Interval	p-value
Age	1.062	1.048-1.076	<0.0001
Age points	1.757	1.548-1.994	<0.0001
Oxygen saturation	0.9411	0.9265-0.9559	<0.0001
Oxygen saturation <92%	2.390	0.4184-3.230	<0.0001
C-reactive protein	1.004	1.003-1.006	<0.0001
C-reactive protein >10mg/l	1.751	0.9945-3.084	0.0523
Creatinine	1.144	1.074-1.219	<0.0001
Creatinine >84µmol/l	1.942	1.378-2.738	0.0002
Risk stratification score	1.218	1.163-1.276	<0.0001
Risk stratification score >22	3.723	2.727-5.083	<0.0001
Risk stratification score >23	4.124	2.991-5.688	<0.0001

TableS3: Hazard ratios for 21-day mortality of the risk stratification score, its components and categorizations.

Predictor	Hazard Ratio	95% Confidence Interval	p-value
Age	1.057	1.044-1.070	<0.0001
Age points	1.701	1.514-1.911	<0.0001
Oxygen saturation	0.9479	0.9337-0.9622	<0.0001
Oxygen saturation <92%	2.097	1.584-2.776	<0.0001

Supplementary Information

C-reactive protein	1.003	1.002-1.005	<0.0001
C-reactive protein >10mg/l	1.927	1.1119-3.320	0.0181
Creatinine	1.121	1.053-1.193	0.0003
Creatinine >84µmol/l	1.761	1.287-2.409	0.0004
Risk stratification score	1.196	1.147-1.248	<0.0001
Risk stratification score >22	3.147	2.340-4.234	<0.0001
Risk stratification score >23	3.595	2.645-4.886	<0.0001

Supplementary Figures:

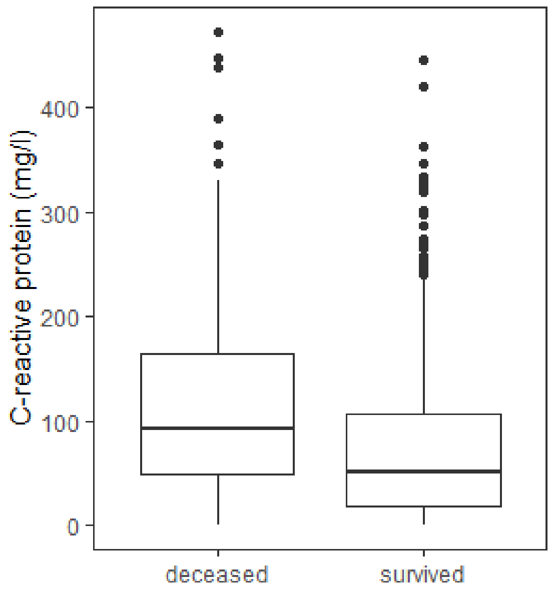

FigureS1: C-reactive protein levels according to 28-day mortality
